# Supplementary material for: Dynamin Inhibitors Prevent the Establishment of the Cytomegalovirus Assembly Compartment in the Early Phase of Infection
Source: Life (Basel). 2021 Aug 25;11(9):876. doi: 10.3390/life11090876 (PMC8469281; doi:10.3390/life11090876)
Supplement: Supplementary file 1 [file life-11-00876-s001.zip › life-1336719-supplementary.pdf]

life

MDPI

Article

Dynamin Inhibitors Prevent the Establishment of the Cytomegalovirus Assembly Compartment in the Early Phase of Infection

Igor Štimac[1], Natalia Jug Vučko[1], Gordana Blagojević Zagorac[1,2], Marina Marcelić[1], Hana Mahmutefendić Lučin[1,2*], and Pero Lučin[1,2]

[1] Department of Physiology and Immunology, University of Rijeka Faculty of Medicine, 51000 Rijeka, Croatia; igor.stimac@uniri.hr (I.Š.); natalia.jug@uniri.hr (N.J.V.); gordana.blagojevic@uniri.hr (G.B.Z.); mmarcelic@uniri.hr (M.M.); hana.mahmutefendic@uniri.hr (H.M.L.); pero@uniri.hr (P.L.)

[2] University North, University Center Varaždin, Jurja Križanića 31b, 42000 Varaždin, Croatia; hmahmutefendic@unin.hr (H.M.L.)

* Correspondence: hana.mahmutefendic@uniri.hr Tel.: (optional; include country code; if there are multiple corresponding authors, add author initials)

1. Supplementary Figures

A

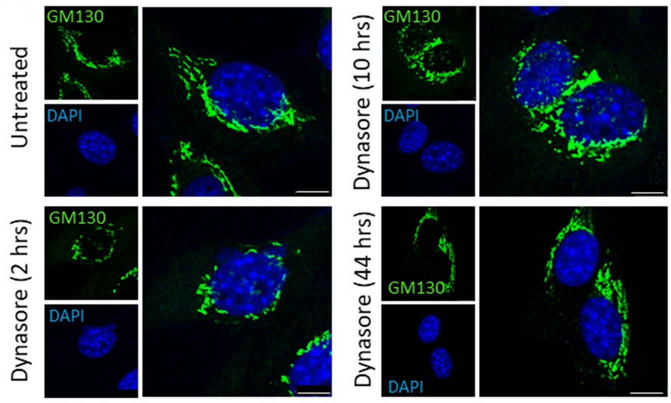

B

Dynasore	Golgi pattern (%)			
	Aa	Ab	B	C
Untreated	62.1± 5.2	28.8± 3.0	7.6± 4.7	0.5± 0.4
2 hrs	29.1± 2.5	63.9± 7.48	7.59± 5.67	0
10 hrs	5.3± 3.56	84.1± 5.7	8.8± 2.4	1.7± 1.0
44 hrs	5.4± 5.6	87.3± 4.7	6.7± 7.8	0

Figure S1. *The effect of Dynasore on Golgi pattern in uninfected Balb3T3 fibroblasts.* Cells plated on coverslips were left untreated or treated with Dynasore (80 µM) for 2, 10, and 44 hrs. (**A**) Representative focal-plane confocal images of cells after staining against GM130 (cis-Golgi; green) and DAPI (blue) visualization of the nuclei. Bars, 10 µm. (**B**) The percentage of cells expressing one of four identified Golgi patterns visualized by anti-GM130 staining. The data represent the mean±SD from three independent experiments.

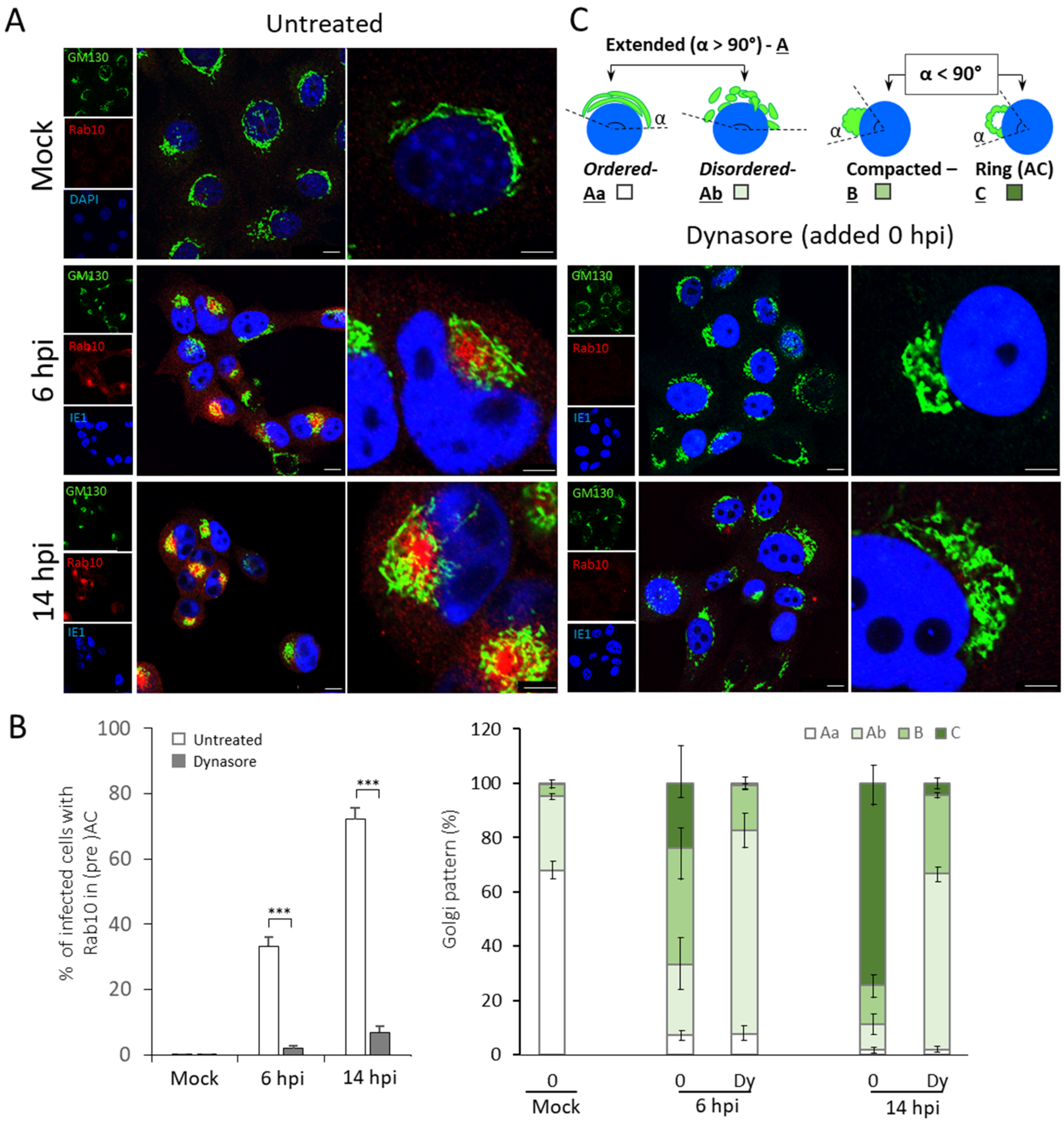

Figure S2. *Dynasore inhibits the establishment of the preAC when added before viral internalization.* (**A**) Cells were plated on coverslips and infected with Δm138-MCMV. Dynasore (80μM) was added together with the virus, or cells were left untreated. At 6, 14, and 48 hrs post-infection (hpi), the cells were fixed, permeabilized, and stained against Rab10 (EE/ERC marker; red fluorescence), GM130 (cis-Golgi; green fluorescence), and IE1 (a nuclear marker of infection; blue fluorescence). In mock-infected cells, the blue color represents the DAPI nuclear staining. Scale bar is equal to 10 μm in smaller and 5 μm in larger magnifications. Representative focal-plane images of three independent experiments are shown. (**B**) The percentage of infected cells (IE1 positive) expressing juxtanuclear accumulation of Rab10 (Rab10 in the preAC) and one of four Golgi patterns in untreated and dynasore-treated cells. The right panel presents the relative distribution of different Golgi patterns visualized by GM130. Data represent the mean values, and error bars show standard deviations. The significance to the untreated samples of the same kinetics was determined by the Student's t-test (***$P < 0.001$). (C) Schematic presentation of Golgi patterns.

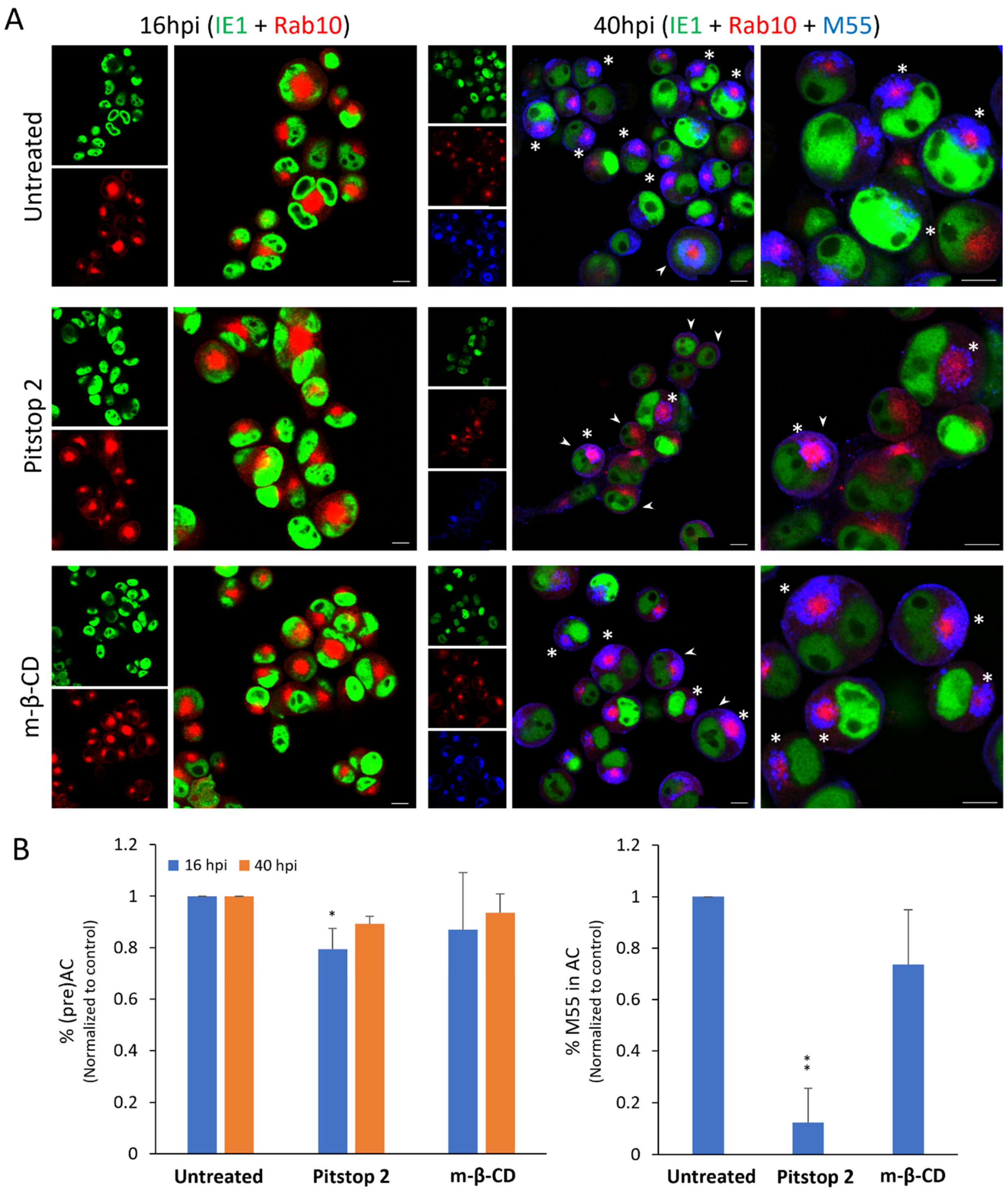

Figure S3. *The effect of Pitstop and methyl-β-cyclodextrin on the establishment of the preAC and development of the AC.* Δm138-MCMV infected cells were treated with Pitstop 2 (50 μM, added at 4 hpi), or β-methyl-cyclodextrin (7,5 mM, added with the virus) and incubated in the presence of inhibitors up to 16 and 40 hpi. (**A**) Double and triple immunofluorescence images of 16 and 40 hrs infected cells, respectively, stained against IE1 (green), Rab10 (red), and M55/gB (blue). Asterisks point to M55 in the AC and arrowheads to M55 at the cell surface. Bars, 10 μm. Representative images of three independent experiments are shown. (**B**) Quantification of the impact of inhibitors on establishing the preAC indicated by Rab10 accumulation (left panel) and M55 loading into the outer AC (right panel). Results on graphs are presented as normalized to control [(%Rab10$_{Inh.}$ /%Rab10$_{Ø}$) or (%M55-AC$_{Inh.}$ /%M55-AC$_{Ø}$)]. Only IE1^{+} cells were analyzed. The data represent the mean±SD from 3 independent experiments. The significance to untreated samples from the same kinetics was determined using the Student's t-test (**P < 0.01, *P < 0.05).

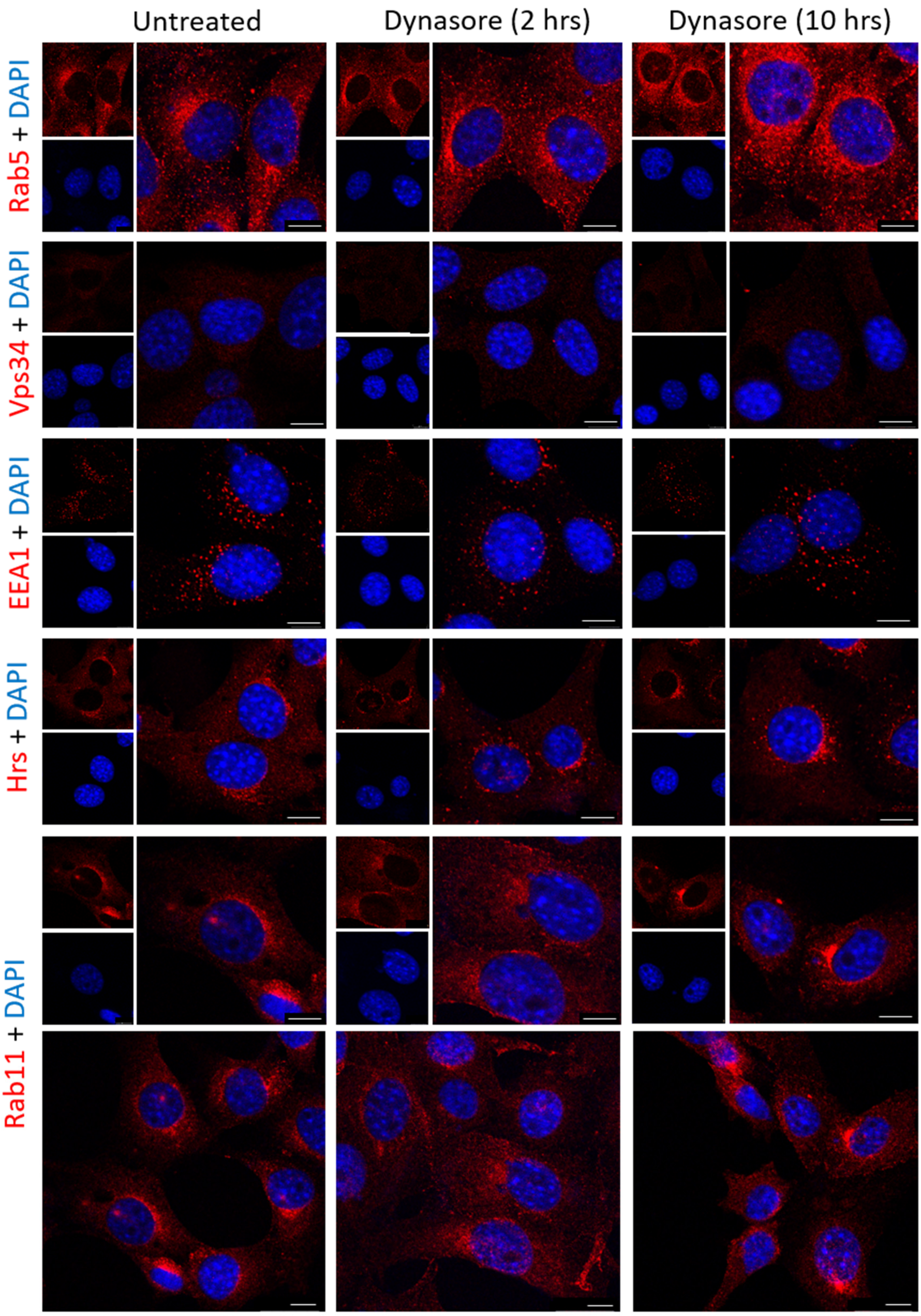

Figure S4. *The effect of Dynasore on EE/ERC markers in uninfected Balb3T3 fibroblasts.* Cells plated on coverslips were left untreated or treated with Dynasore for 2 hrs and 10 hrs and proceeded for immunofluorescence staining of Rab5, Vps34, EEA1, Hrs, and Rab11 (red). Nuclei were visualized by DAPI (blue). Representative focal-plane images of three independent experiments are shown. Bars, 10 µm.

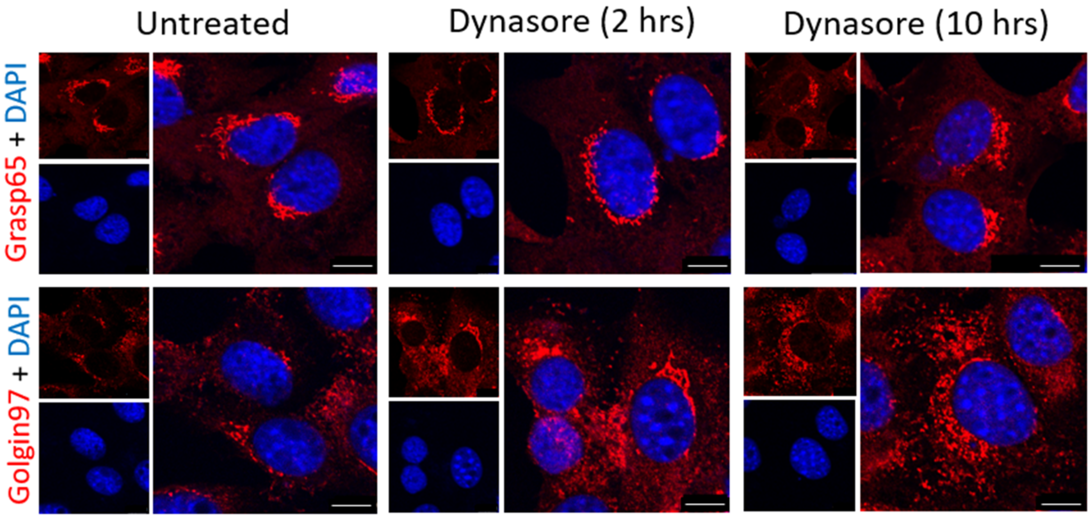

Figure S5. *The effect of Dynasore on Golgi markers in uninfected Balb3T3 fibroblasts*. Cells plated on coverslips were left untreated or treated with Dynasore for 2 and 10 hrs and proceeded for immunofluorescence staining of Grasp65 and Golgin97 (red). Nuclei were visualized by DAPI (blue). Representative focal-plane images of two independent experiments are shown. Bars, 10 µm.

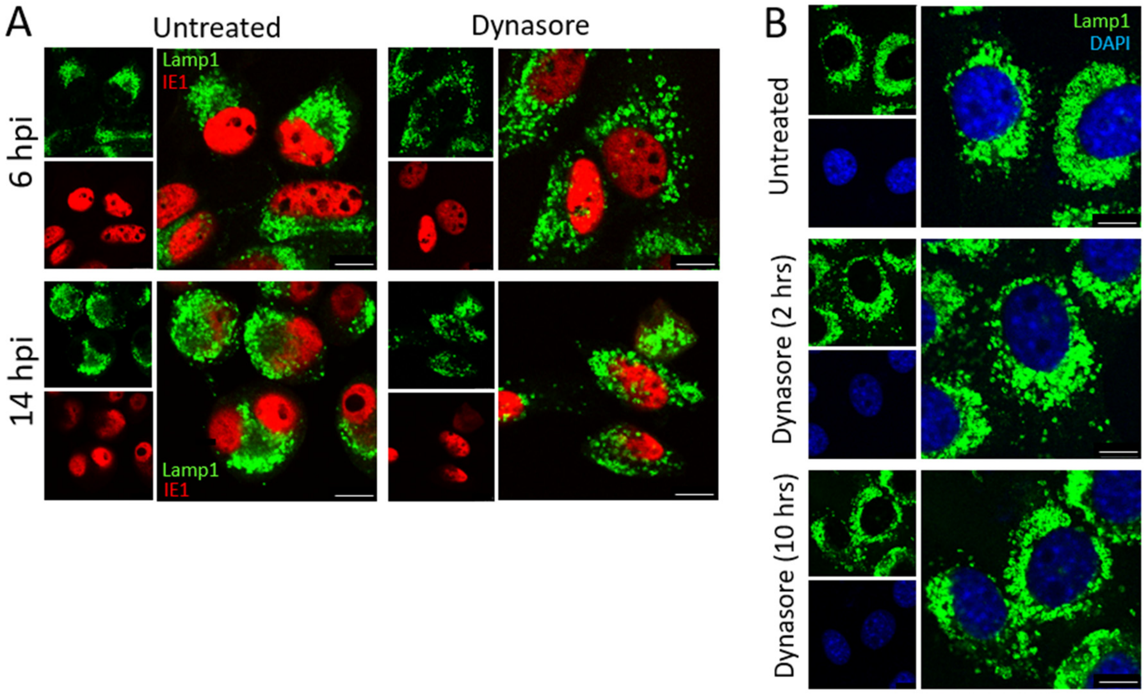

Figure S6. *The effect of Dynasore on Lamp1 membranes in Balb3T3 fibroblasts*. Δm138-MCMV infected cells were treated with dynasore (80 µM) at 4 hpi or left untreated and were proceeded for immunofluorescence 6 hpi and 14 hpi. (**A**) Immunofluorescence images of infected cells: Lamp1 (red) and IE1 (red). (**B**) Immunofluorescence images of uninfected cells: Lamp1 (green) and DAPI (blue) after 0, 2, and 10 hrs of dynasore treatment. Results are representative of two independent experiments. Bars, 10 µm.

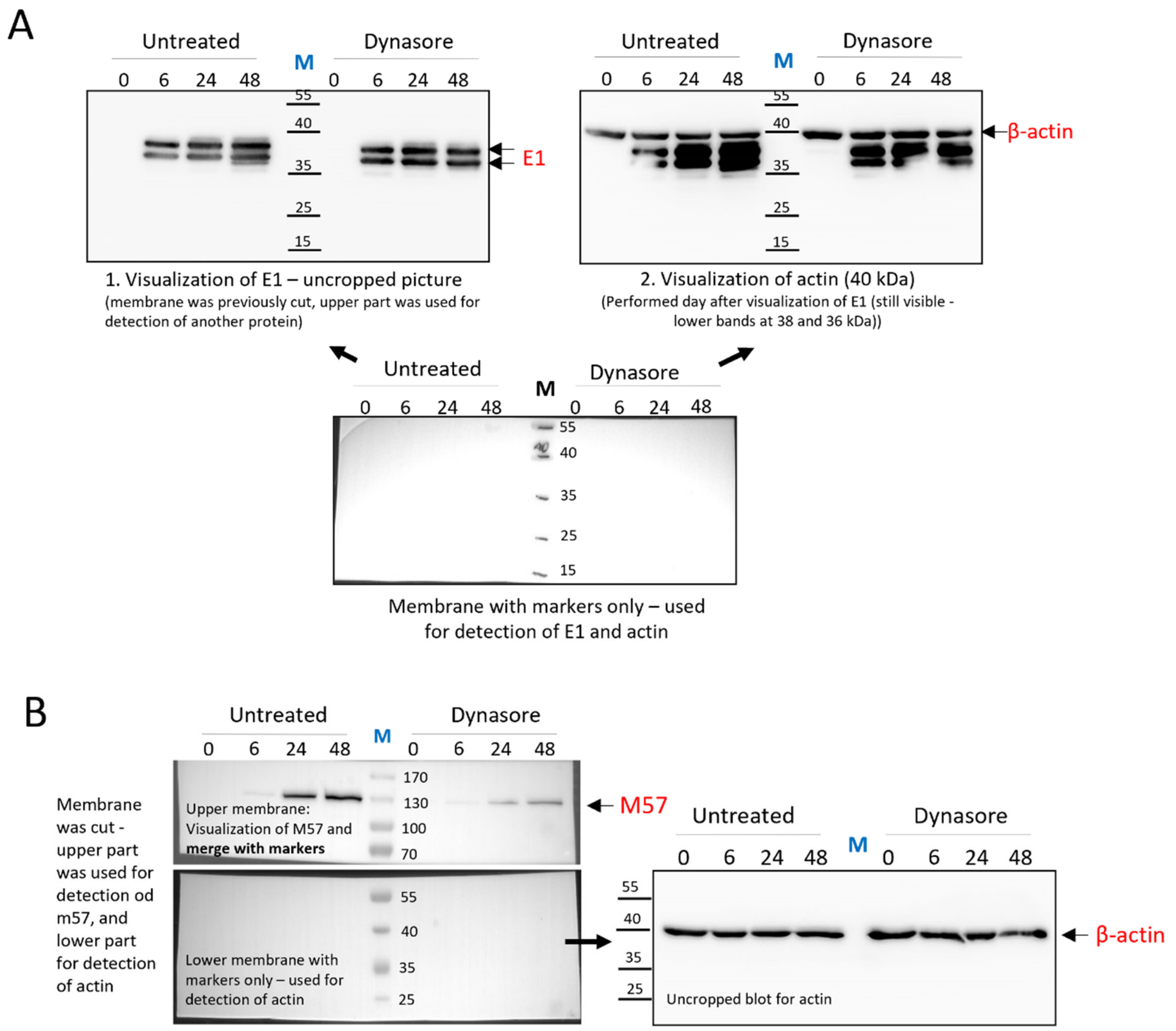

Figure S7. *Uncropped Western blots and membranes with markers used as a representative Western blot in Figure 8 of the manuscript.* (**A**) Analysis of E1 expression: Membrane for detection of E1 was cut. The lower part (shown here) was used to detect E1 and actin, and the upper part for detection of another protein (not shown here). The E1 (38 and 36 kDa; left) was detected first, followed by detecting actin (42 kDa) on the same membrane. The membrane only with markers is shown below. (**B**) Analysis of M57 expression: the membrane was cut, the upper part was used to detect M57 (130 kDa), and the lower part for detection of actin (left panel). M57 is presented merged with markers (upper part of left panel), and detection of β-actin is shown separately (right panel).

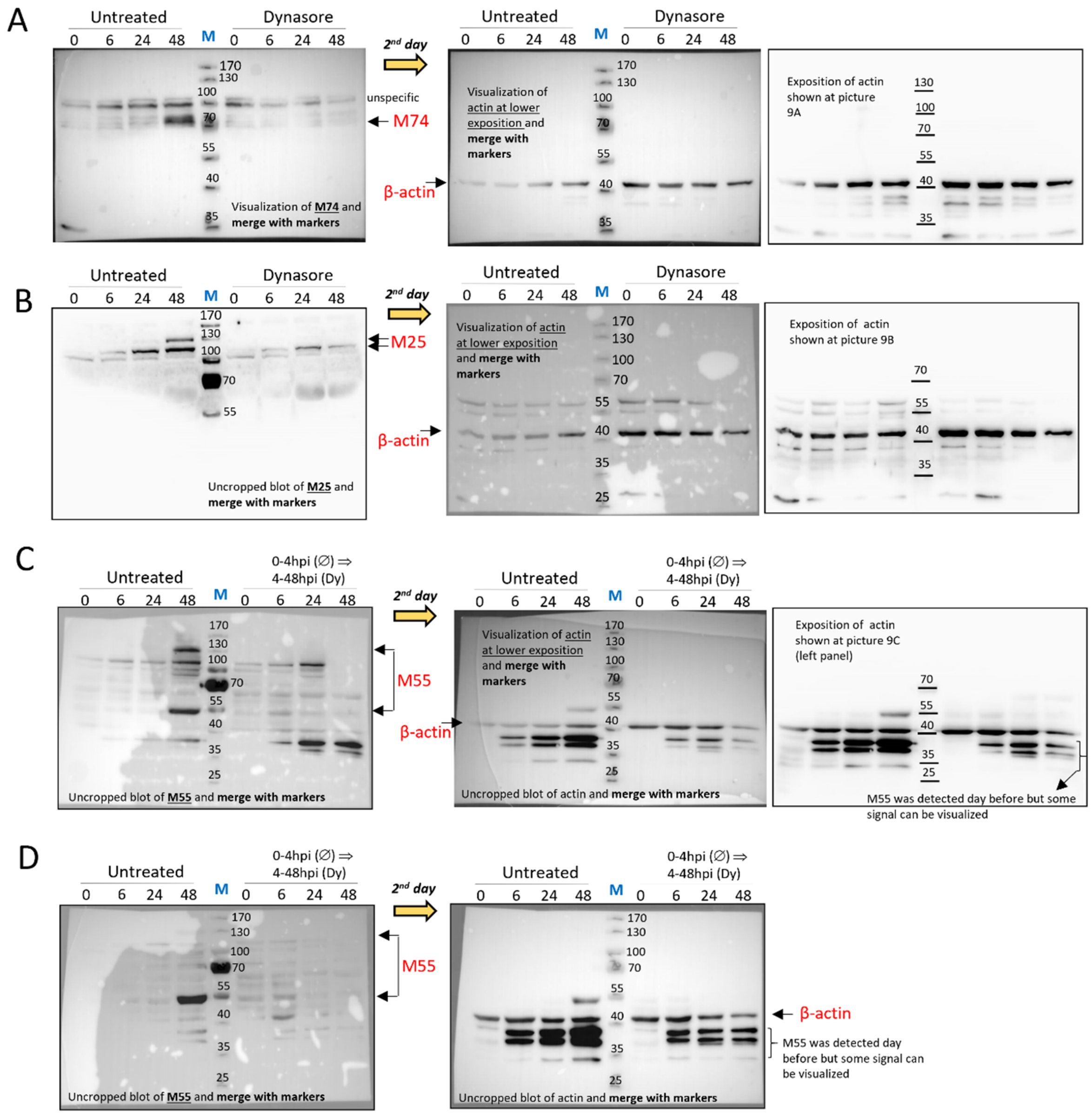

Figure S8. *Uncropped Western blots and membranes with markers used as a representative Western blot in Figure 9 of the manuscript.* Uncropped membranes merged with MCMV late proteins are shown: M74 (A, left panel), M25 (B, left panel), M55 – untreated + dynasore (4-48 hpi) (C, left panel), and M55 – untreated + dynasore (4-14 hpi) (D, left panel). Detection of β-actin was performed the next day for all experiments. Membranes that show actin signals merged with markers are presented in adjacent panels. When actin was merged with markers only at a low exposition, additional pictures of actin presented in final figures (9A, 9b, and 9C) are shown (right).

2. Supplementary Tables

Table S1. The effect of dynasore on the expression of markers of cellular compartments (*Related to data presented in Figures 4-6, and Figures S4-S6).*

Marker of cellular compartment	Untreated	Uninfected		MCMV infection			
		2 hrs	10 hrs	6 hpi		14 hpi	
		Untreated	Dynasore	Untreated	Dynasore	Untreated	Dynasore
				**EARLY ENDOSOMES**			
**Rab5**	Perinuclear and cytoplasmic vesicles	Perinuclear and partially dispersed vesicles	Perinuclear and cytoplasmic vesicles	preAC/ Perinuclear	Cytoplasmic	preAC	Cytoplasmic,
**Vps34**	Not detected	Not detected	Not detected	preAC	Diffuse cytoplasmic	preAC	Diffuse cytoplasmic
**EEA1**	Cytoplasmic vesicles	Cytoplasmic vesicles/ vacuoles	Cytoplasmic vesicles/ vacuoles	Cytoplasmic and preAC	Cytoplasmic vacuoles	preAC	Cytoplasmic vacuoles
**Hrs**	Perinuclear and cytoplasmic vesicles	Perinuclear and dispersed cytoplasmic vesicles	Perinuclear and dispersed cytoplasmic vesicles	preAC and cytoplasmic	Diffuse cytoplasmic	preAC	Diffuse cytoplasmic
				**EARLY ENDOSOMES TO ENDOSOMAL RECYCLING COMPARTMENT**			
**Rab10**	Not detected	Not detected	Not detected	preAC and cytoplasmic	Not detected	preAC	Not detected
				**ENDOSOMAL RECYCLING COMPARTMENT**			
**Rab11**	Perinuclear	Dispersed in cytoplasm	Perinuclear and pericentriolar	preAC	Diffuse	preAC	Concentrated in the inner preAC and subplasmalemmal
**Arf6**	Not detected	Not detected	Not detected	Not detected	Not detected	preAC	Not detected
				**GOLGI COMPARTMENT**			
**Grasp65 (ERGIC-*cis*-Golgi)**	Extended Golgi cisternae	Disordered and vacuolized Golgi	Disordered and vacuolized Golgi	Outer AC, condensed, or disordered Golgi	Disordered, or some condensed Golgi	Disordered as outer AC or condensed	Dispersed (fragmented), peripheral, ER
**GM130 (*cis*-Golgi)**	Extended Golgi cisternae	Disordered and vacuolized Golgi	Disordered and vacuolized Golgi	Outer AC, condensed, or disordered Golgi	Mostly disordered Golgi	Dominantly the outer AC	Mostly disordered Golgi, some condensed
**Golgin97 (*trans*-Golgi)**	Extended Golgi cisternae and cytoplasmic vesicles	Disordered, vacuolized, and cytoplasmic vesicles	Disordered in the cytoplasm. Visible in periphery	Disordered, and Outer and inner AC	Disordered and Condensed Golgi. Periphery.	Outer and inner AC and periphery	Dispersed periphery, rarely outer preAC
				**LATE ENDOSOMES**			
**Lamp1**	Cytoplasmic vesicles/ vacuoles	Cytoplasmic vacuoles	Cytoplasmic vacuoles	Cytoplasmic vacuoles	Cytoplasmic vacuoles	Cytoplasmic vacuoles	Cytoplasmic vacuoles

Δm138-MCMV infected, and uninfected Balb 3T3 cells were treated with Dynasore (80 μM). Infected cells were treated at 4 hpi, and uninfected cells were treated with the inhibitor for the same period as infected cells. After immunofluorescence staining against indicated marker proteins, the cells were analyzed as explained in Figs. 4-6, Figs. S4-S6 and Material and Methods.
